# Supplementary material for: Three‐dimensional chromatin re‐organization during muscle stem cell aging
Source: Aging Cell. 2023 Feb 2;22(4):e13789. doi: 10.1111/acel.13789 (PMC10086523; doi:10.1111/acel.13789)
Supplement: Supplementary file 1 — Appendix S1 [file ACEL-22-e13789-s001.pdf]

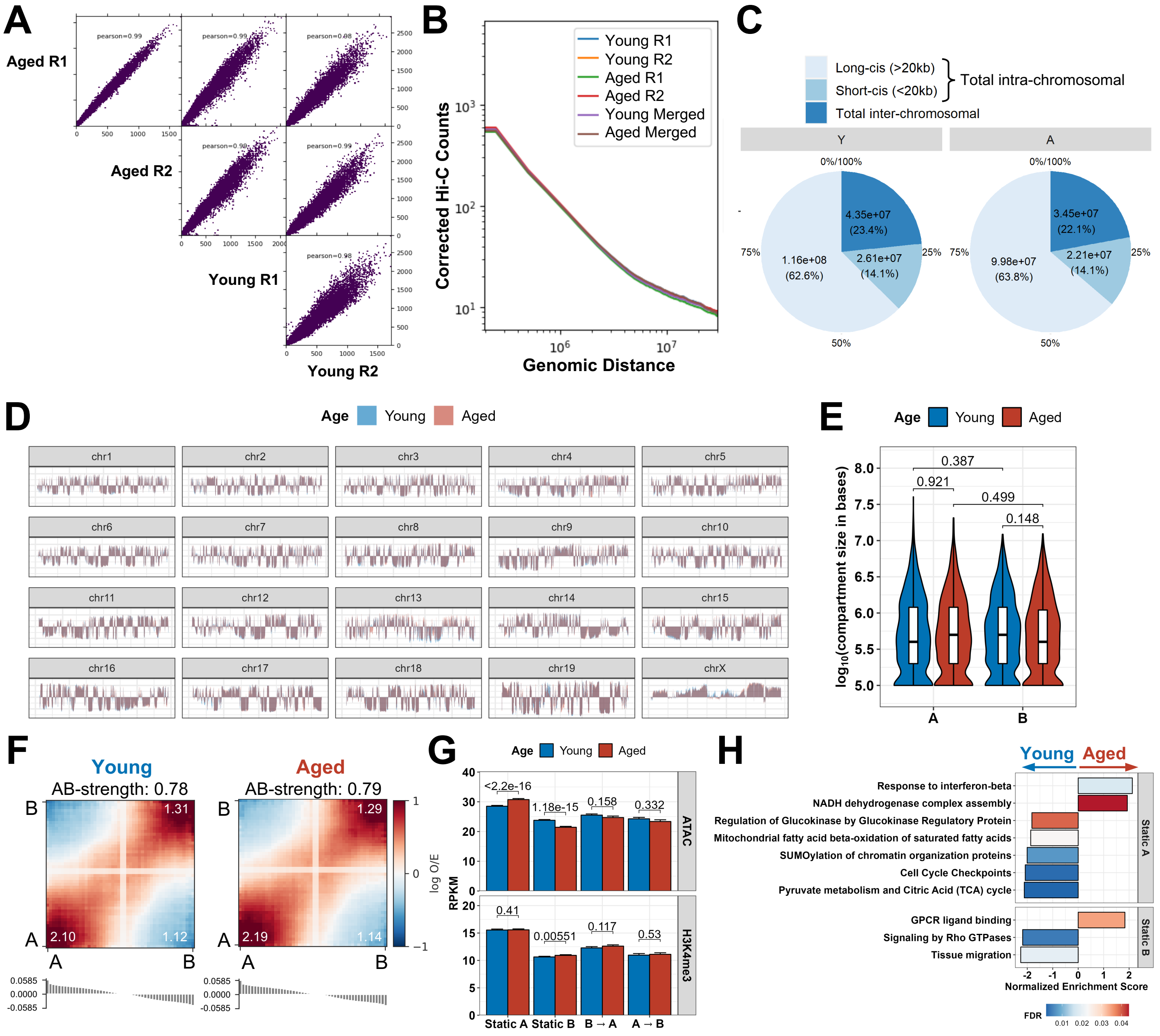

Supplementary Figure 1

Supplementary Figure 1. Quality control assessments of Hi-C datasets from young and aged muscle stem cells. A) Pairwise Pearson correlation scatterplots of observed/expected counts in young and aged replicates at 250 kb resolution. **B)** Number of contacts at 250 kb resolution between loci of increasing genomic distance for each young and aged replicate compared with the corresponding merged replicates. **C)** Pie chart of the number of reads that map to inter- and intra-chromosomal interactions at 100 kb resolution. Long-cis and short-cis interactions refer to interactions on the same chromosome between loci separated by >20 kb and <20 kb, respectively. **D)** Overlapped first principal components of the Pearson correlation matrix of young and aged MuSC contact maps. **E)** Sizes of A/B compartments in young and aged MuSCs in $\log_{10}$(bases). **F)** Saddle plots of genome-wide A/B compartment enrichment. Eigenvector percentiles used to calculate compartment enrichment are shown below. **G)** Average RPKM signals per compartment switching region of ATAC-Seq and H3K4me3 datasets between aged and young MuSCs. **H)** Enriched GO and Reactome terms for genes in chromatin compartment groups identified through GSEA. All statistical comparisons are unpaired Mann-Whitney U-tests.

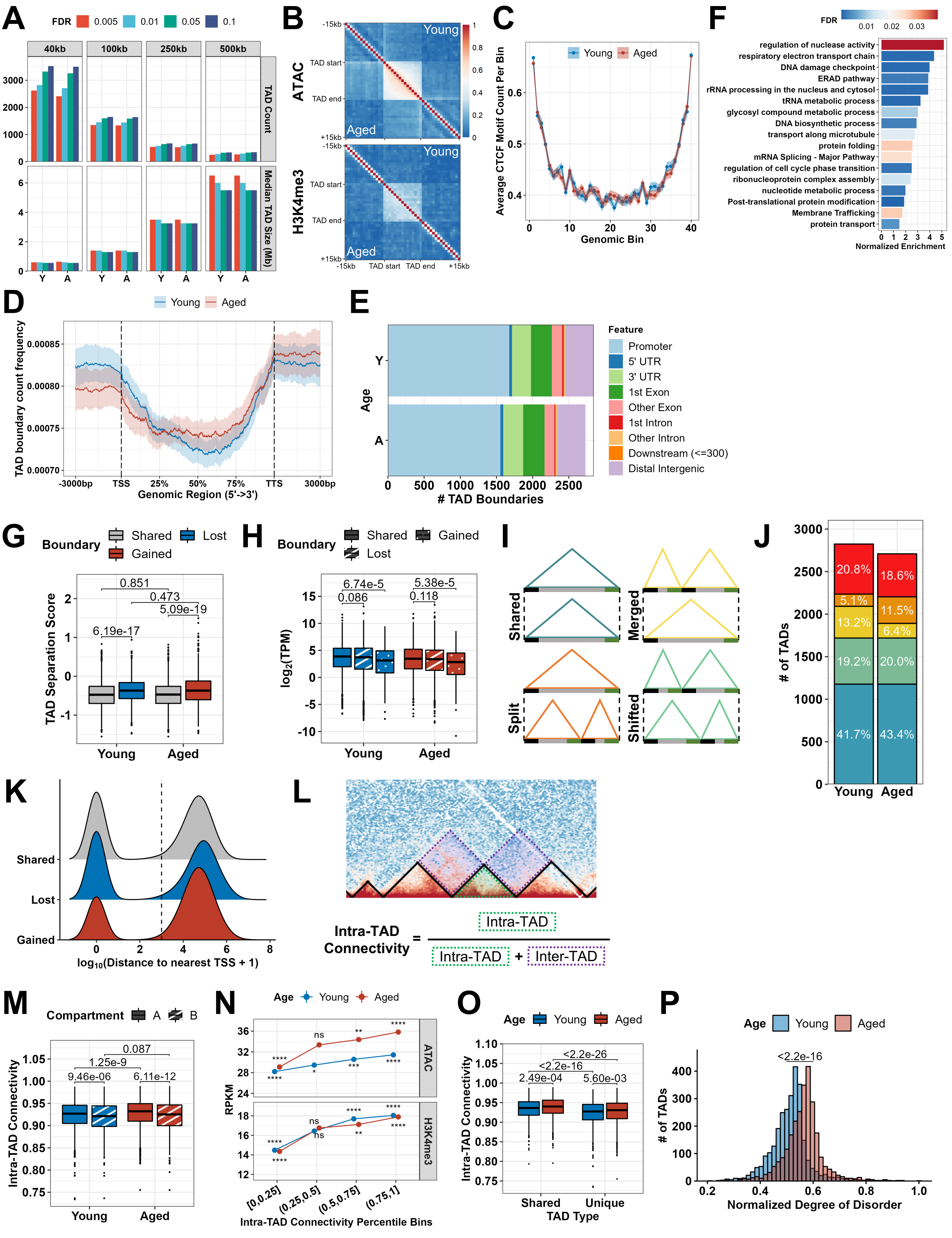

Supplementary Figure 2

Supplementary Figure 2. Definition and annotation of Topologically Associated Domains in young and aged muscle stem cells. A) Optimization of the contact map resolution and FDR parameter used in HiCExplorer (hicFindTads) for finding TADs. **B)** Pearson correlation matrices of ATAC and H3K4me3 signals inside and within 15 kb of TAD domains. **C)** Distribution of CTCF motif enrichment across TAD domains. **D)** Comparison of the distribution of TAD boundaries across gene bodies. **E)** Annotation of TAD boundaries. **F)** Representative GO and Reactome terms for housekeeping genes enriched at shared TAD boundaries. **G)** TAD separation scores and **H)** gene expression at lost, gained, and shared TAD boundaries. **I)** Cartoon showing definitions of rearranged TAD types in young MuSCs (top) relative to aged MuSCs (bottom). **J)** Proportions of TAD rearrangements. **K)** Distance in basepairs of TAD boundaries to the nearest expressed transcription start site. A vertical line is drawn at 1kb, representing the defined region for promoters. **L)** Diagram of intra-TAD connectivity. The mean read counts per intra-TAD interaction are divided by the sum of mean read counts for all interactions per TAD. Intra-TAD regions are highlighted in green while inter-TAD regions are highlighted in purple. **M)** Comparison of intra-TAD connectivity by compartment. **N)** ATAC and H3K4me3 signals in RPKM (mean +/- SEM) against binned intra-TAD connectivity percentiles. Statistical comparisons are pairwise Mann-Whitney comparisons between each percentile bin against the mean of all bins within each signal type and age group. (p-values: *<0.05, **<1e-2, ****<1e-3, ****<1e-4). **O)** Comparison of intra-TAD connectivity between unique and shared TADs. Statistical comparisons are unpaired Mann-Whitney U-tests. **P)** Degree of disorder distributions for young and aged TADs. The statistical comparison is an unpaired Mann-Whitney U-test.

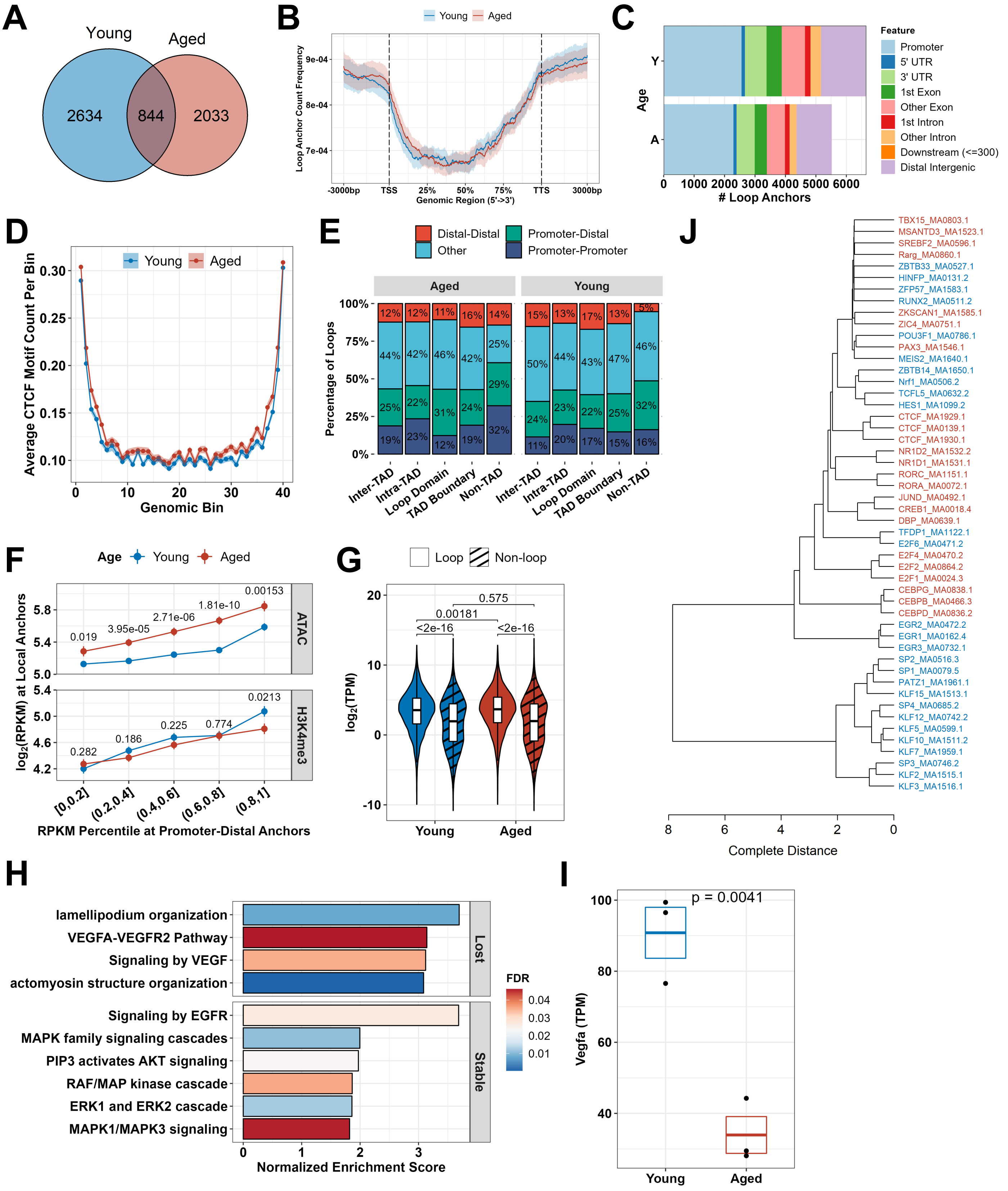

Supplementary Figure 3

Supplementary Figure 3. Characterization of genomic interactions and chromatin loop domains in young and aged muscle stem cells. A) Venn diagram of chromatin loops. **B)** Comparison of the distribution of loop anchors across gene bodies. **C)** Annotation of loop anchors. **D)** Distribution of CTCF motif enrichment between loop anchors. **E)** Classification of loop anchors across loop types. **F)** H3K4me3 and ATAC-seq signal strengths in RPKM (mean +/- SEM) at anchors of promoter-distal loops. Statistical comparisons are unpaired Mann-Whitney U-tests between age groups within each bin. **G)** Comparison of gene expression between or outside of chromatin loop anchors. Statistical comparisons are unpaired Mann-Whitney U-tests. **H)** GO and Reactome terms enriched in loops that are stable or lost with aging. **I)** Expression of *Vegfa* in TPM. Statistical comparison is a Student's t-test. **J)** Hierarchical clustering of inferred differentially bound transcription factors by overlap of TF binding sites. Motif names are colored by the direction of enrichment (Young—blue; Aged—red).

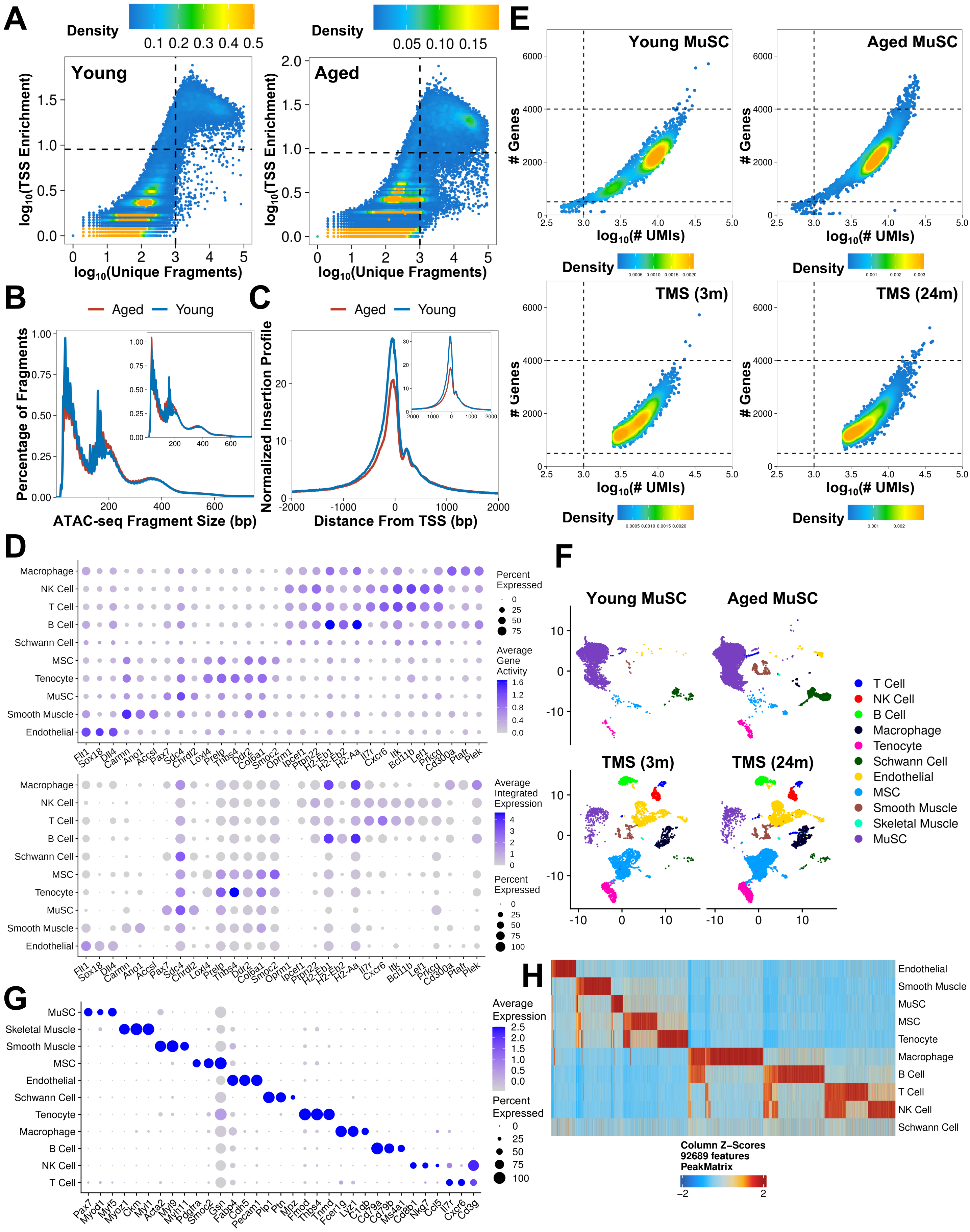

Supplementary Figure 4

Supplementary Figure 4. Quality assessments of single young and aged muscle stem cell multi-omic datasets. A) QC plots of TSS enrichment against the $\log_{10}$(number of unique fragments) per cell across samples. **B) and C)** Density plots of **B)** fragment size and **C)** enrichment of insertion sites around TSS sites. Insets show data specific for MuSCs. **D)** Dot plots of gene activity scores (top) and integrated single cell RNA (scRNA) gene expression (bottom) for marker genes in identified cell type clusters. **E)** QC plots of previously collected MuSC scRNA datasets and age-matched datasets from Tabula Muris Senis (TMS). **F)** UMAP embeddings of scRNA data colored by cell type and split by dataset. **G)** Dot plot of marker gene expression in integrated scRNA datasets. **H)** Row-scaled heatmap of differentially accessible peaks (FDR <= 0.01 & Log2FC >= 1) in each cell type cluster.

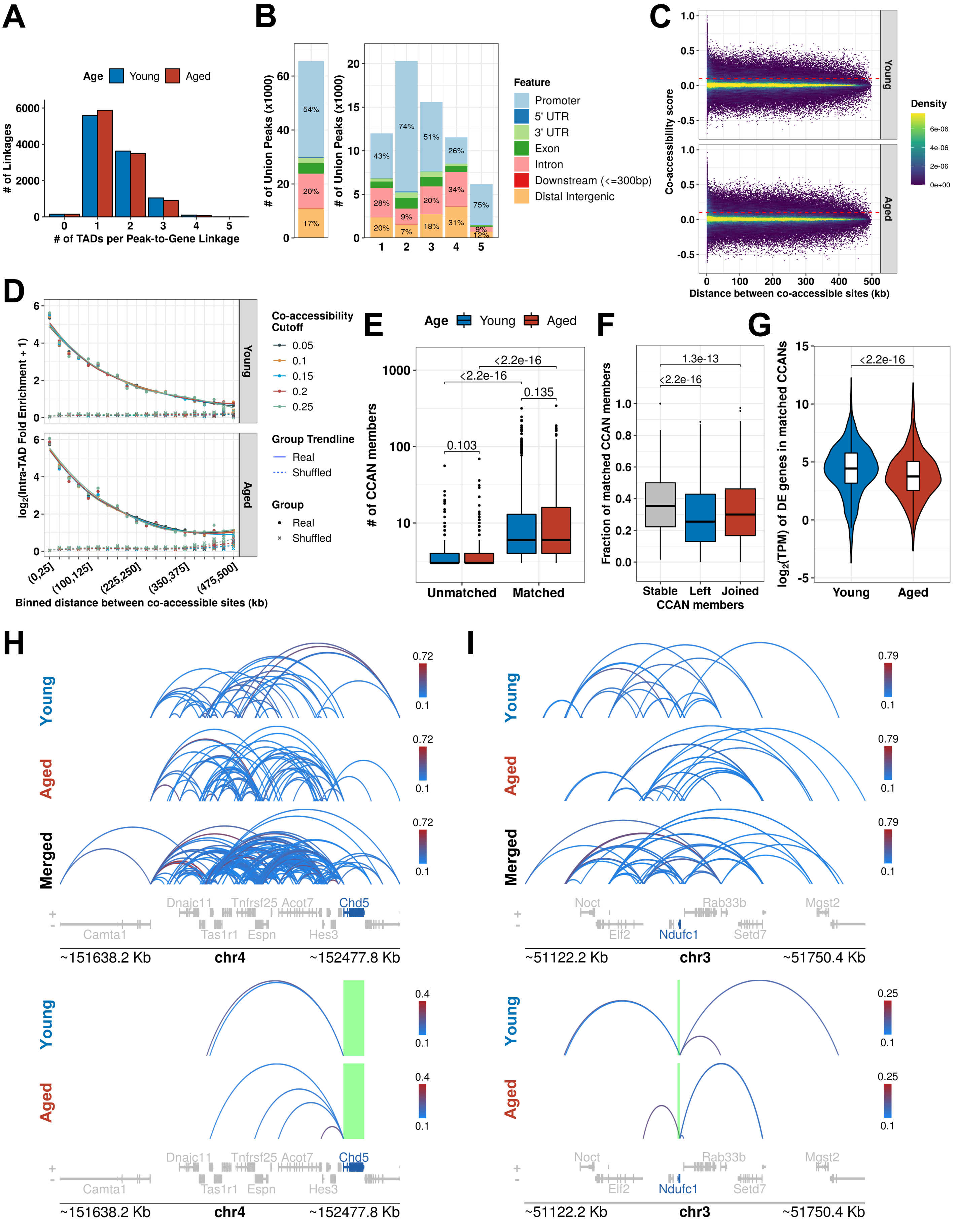

Supplementary Figure 5

Supplementary Figure 5. Mapping gene expression regulatory networks in single young and aged muscle stem cells. A) Histogram of the number of TADs per peak-to-gene linkage. **B)** Annotation of union MuSC peakset and peaks in each peak-to-gene cluster. **C)** Scatter plot of all identified co-accessible sites using Cicero. The minimum co-accessibility score threshold (0.1) used to filter sites is shown in red. **D)** Intra-TAD enrichment (log2 odds ratio) of linked peaks compared to randomly linked distance-matched peaks across TADs across different co-accessibility thresholds. **E)** Numbers of sites in matched and unmatched CCANs. **F)** Average fractions of CCAN members that leave, join, or remain in matched CCANs during aging. **G)** Expression of differentially expressed genes in matched CCANs. **H) and I)** Representative plots showing altered connectivity within young and aged CCANs relative to CCANs identified in merged young and aged data for the **H)** *Chd5* and **I)** *Ndufc1* loci. The top group of plots shows the CCAN containing each gene. The bottom group shows zoomed-in connections in each CCAN that lie in each gene body, which is marked by a green rectangle. Statistical comparisons are unpaired Mann-Whitney U-tests.

Sample	# QC'ed Cells	Median Peaks per Cell	Median Fragments per Cell	Median TSS Enrichment	Median Blacklist Ratio	Median FRiP
Young	10,603	3,144	5,451	25.15	0.0228	0.552
Aged	14,327	5,273	10,016	19.41	0.0230	0.529

Sample	# QC'ed MuSCs	Median Peaks per Cell	Median Fragments per Cell	Median TSS Enrichment	Median Blacklist Ratio	Median FRiP
Young	540	1,825	3,766.5	27.94	0.0250	0.505
Aged	457	3,685	10,350	18.95	0.0236	0.356

Supplementary Table 1. Quality assessment metrics of single cell ATAC datasets.
